# Practical Notes and Formulæ

**Published:** 1888-08

**Authors:** 


### PRACTICAL NOTES AND FORMULÆ.

**A Hypnotic for Use in Alcoholism.**—Mann, of Brooklyn, in reporting good results in the treatment of alcoholism, states that he found the following a useful hypnotic. At night 2 tablespoonsful were given;

℞. Tr. opii deod..............................

Ext hyoscy. fld ..............................aa ʒ j,

Chloral hydrat..............................

Pot. bromid..............................aa ʒ j,

Tr. capsici..............................ʒ ss,

Tr. aconit. rad..............................*m* v,

Aq. menth. pip....................q. s. ad. fl. ℥ iv.

M. —*Brooklyn Medical Journal.*

**For Vomiting in Cholera Infantum.**—*Hughes*:

℞. Bismuth subnit..............................gr. x,

Mucil. acaciæ..............................ʒ ss,

Acid carbolici..............................gr. ss,

Tinct. opii deodorat..............................gtt. j,

Mist. cretæ..............................ʒ ss.

M. S.—To be given every two hours, for a child one to two years old.

**To Stop Toothache**—Gesell Fels makes the following mixture, which is an oily liquid, and introduced into the tooth cavity has proved very effective:

℞. Camphor..............................gr. lxxv,
Chloral hydrati..............................gr. lxxv,
Cocaini muriat..............................gr. xv.

—*Therapeutic Gazette.*

**Cracked nipples** are treated with great success by Pinard, as follows: As soon as there are any appearances of cracks, or even tenderness of the nipples, a compress folded in four and steeped in boracic-acid solution, three or four per cent., is applied. Oil silk is placed over the compress to prevent evaporation. Over this a layer of cotton wadding, and the whole secured by a bandage.—*American Practitioner and News.*

**Cocaine Cotton.**—Mr. K. Eller describes in the *Druggist's Circular*, a very easy, neat and successful way whereby to obtain the effect of cocaine by combining it with absorbent cotton. As thus prepared, it may be applied in a variety of ways to assuage pain and bring relief to those suffering. It requires the suggestion merely in order to insure its ready preparation by an ordinarily skillful pharmacist: for instance, a given weight of absorbent cotton, a certain quantity of solution of cocaine of definite strength, and the requisite skill, and a cocaine cotton of exact cocaine value will be the result. A three per cent. solution of cocaine is a good average strength to work with, and on this basis the following formulæ are suggested unless an exception be noted:

COCAINE COTTON.

℞. Solution cocaine, three per cent. . . . . . . . . . . . . . . . . . ℥ j,
Absorbent cotton . . . . . . . . . . . . . . . . . . . . . . . . . . . . . . . ʒ j.

Thoroughly saturate the cotton, carefully dry the same in a current of warm air, and card the cotton to restore it to its former appearance.

COCAINE COTTON WITH MORPHINE.

(*For toothache.*)

℞. Solution cocaine, three per cent. . . . . . . . . . . . . . . . . ℥ j,
Sulphate of morphine . . . . . . . . . . . . . . . . . . . . . . . . gr. xii,
Absorbent cotton . . . . . . . . . . . . . . . . . . . . . . . . . . . . ℥ j.

Dissolve the morphine in the cocaine, and proceed as above.

To be used by introducing a small piece of cotton into the cavity of the tooth. It can also be used for earache by moistening a suitable piece, with a few drops of water, laudanum, or alcohol, and carefully placing it in the ear-passage.

BORATED COCAINE COTTON.

(*For burns and scalds.*)

℞. Cocaine solution, two per cent. . . . . . . . . . . . . . . . . ℥ i,
Boracic acid . . . . . . . . . . . . . . . . . . . . . . . . . . . . . . . gr. xxx,
Glycerin . . . . . . . . . . . . . . . . . . . . . . . . . . . . . . . . . . ʒ j,
Carbolic acid . . . . . . . . . . . . . . . . . . . . . . . . . . . . . . gr. xx,
Absorbent cotton . . . . . . . . . . . . . . . . . . . . . . . . . . . . ℥ j.

Dissolve the boracic acid in the glycerin and cocaine solution, add the carbolic acid, and proceed as in making plain cocaine cotton.

Very useful in the treatment of burned and scalded surfaces, and also in troublesome chafing, where pain and persistent inflammation are attendants.

This list is capable of very large extension. Only this short suggestion is needed to enable the pharmacist to carry his experiments to the limit of wish or convenience.—*Therapeutic Gazette.*

**Antipyretics.**—Wholesale prices of—

| | Per ounce. |
|---|---|
| Sulphate of quinine of the best makers at about | $ 40 |
| Tartrate of chinoline | 70 |
| Resorcin | 20 |
| Salicylic acid, patented | 30 |
| Kairin, patented | 2 00 |
| Antipyrin, patented | 1 25 |
| Sulphate of thalin, patented | 1 75 |
| Antifebrin, 30c. per ounce, or as acetanilide | 15 |
| Salol, patented | 40 |

